# Measurement of the migration of a focal knee resurfacing implant with radiostereometry: An experimental study

**DOI:** 10.3109/17453674.2013.869654

**Published:** 2014-02-25

**Authors:** Olof Sköldenberg, Thomas Eisler, André Stark, Olle Muren, Nicolas Martinez-Carranza, Leif Ryd

**Affiliations:** ^1^Department of Orthopaedics at Danderyd Hospital and Karolinska Institutet, Department of Clinical Sciences at Danderyd Hospital (KIDS), Stockholm; ^2^Department of Orthopaedics, Karolinska University Hospital, Stockholm and Karolinska Institutet, Department of Clinical Sciences, Intervention and Technology (CLINTEC), Stockholm; ^3^Episurf Medical AB, Stockholm, Sweden.

## Abstract

**Background and purpose:**

Articular resurfacing metal implants have been developed to treat full-thickness localized articular cartilage defects. Evaluation of the fixation of these devices is mandatory. Standard radiostereometry (RSA) is a validated method for evaluation of prosthetic migration, but it requires that tantalum beads are inserted into the implant. For technical reasons, this is not possible for focal articular resurfacing components. In this study, we therefore modified the tip of an articular knee implant and used it as a marker for RSA, and then validated the method.

**Material and methods:**

We modified the tip of a resurfacing component into a hemisphere with a radius of 3 mm, marked it with a 1.0-mm tantalum marker, and implanted it into a sawbone marked with 6 tantalum beads. Point-motion RSA of the “hemisphere bead” using standard automated RSA as the gold standard was compared to manual measurement of the tip hemisphere. 20 repeated stereograms with gradual shifts of position of the specimen between each double exposure were used for the analysis. The tip motion was compared to the point motion of the hemisphere bead to determine the accuracy and precision.

**Results:**

The accuracy of the manual tip hemisphere method was 0.08–0.19 mm and the precision ranged from 0.12 mm to 0.33 mm.

**Interpretation:**

The accuracy and precision for translations is acceptable when using a small hemisphere at the tip of a focal articular knee resurfacing implant instead of tantalum marker beads. Rotations of the implant cannot be evaluated. The method is accurate and precise enough to allow detection of relevant migration, and it will be used for future clinical trials with the new implant.

We have developed a double-coated monobloc articular resurfacing metal implant, with a small peg for primary fixation, to treat localized, full-thickness articular cartilage defects (Manda et al. 2011, Martinez-Carranza et al. 2013). The osseointegration of this implant after 6 and 12 months has been evaluated in animal models by several authors, with promising but varied results (Kirker-Head et al. 2006, Custers et al. 2009, Custers 2010). However, it has not yet been studied in humans. With radiostereometric analysis (RSA), it is possible to obtain highly accurate 3D measurements from calibrated stereoradiographs. By performing repeated measurements over time, implant migration can be quantified and loosening predicted with high sensitivity (Kärrholm et al. 1994, Ryd et al. 1995). The method requires the insertion of tantalum markers into the skeleton and the implant. Marking of an implant requires modification of the implant design, but it is not feasible for custom-made articular focal knee implants. To our knowledge, no other RSA study has been published on these types of components. The aim of this study was to validate point-motion RSA when applied to a new articular resurfacing metal implant used for focal cartilage knee injuries.

## Material and methods

### Implant

**Figure 1.**
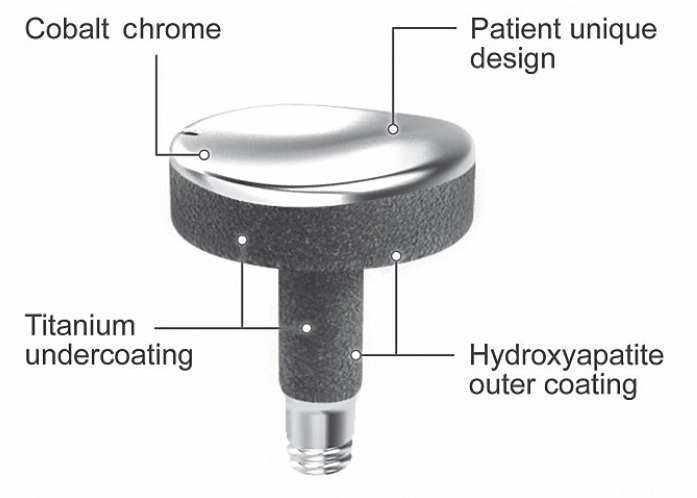
The Episeal articular resurfacing implant.

The Episealer (Episurf AB, Stockholm, Sweden) is a small patient-specific implant designed and specifically crafted for each procedure (Figure 1). The implant is made of a chrome-cobalt alloy (CrCo), the articular surface shape of which is based on preoperative magnetic resonance imaging (MRI). The implant is adapted to each patient’s specific cartilage defect and surrounding joint topography, to recreate a congruent weight-bearing surface. For primary fixation, the monobloc implant has been provided with a peg (3 × 10 mm), which is press-fitted to a centralizing pre-drilled hole (2.8 mm). For permanent fixation, the implant is double-coated with hydroxyapatite on top of a titanium layer (thickness 60 µm) on areas in contact with bone and surrounding cartilage. In animal models, both titanium and hydroxyapatite increase the fixation to bone by osseointegration and adherence to surrounding cartilage (Reigstad et al. 2011).

### Experimental setup

**Figure 2.**
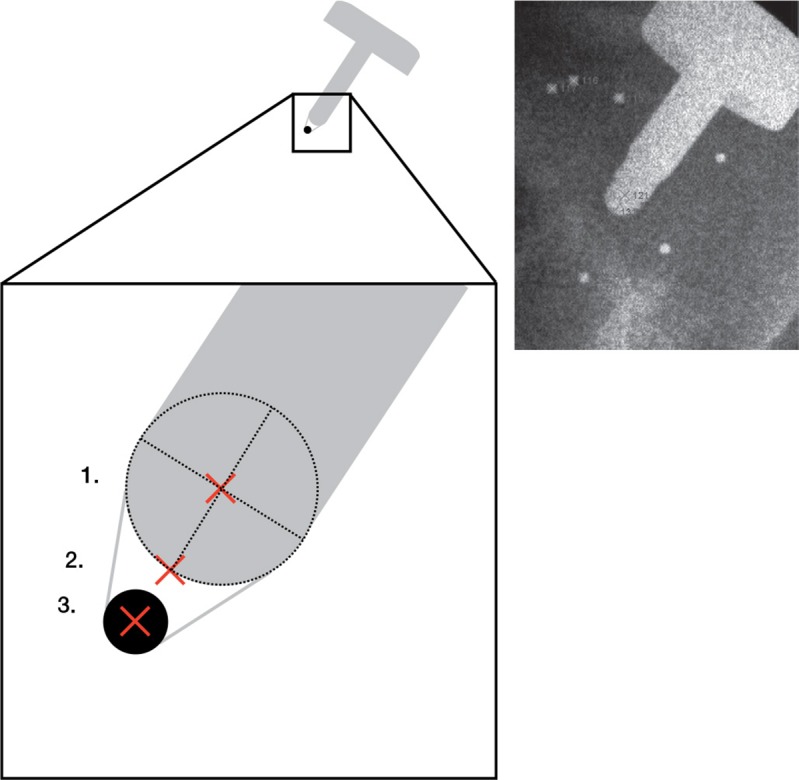
Blown-up schematic illustration of the markers measured on the implant. The distal part of the component was machined into a 3.0-mm hemisphere. A 1.0-mm tantalum marker (black solid circle) was glued to this hemisphere. The markers measured (red crosses) were: (1) manual measurement of the center (dotted circle) of the 3.0-mm hemisphere (hemisphere RSA); (2) manual measurement of the distal tip of the implant (tip RSA); and (3) semi-automated measurement of the tantalum marker (standard RSA). The radiograph shows actual measurements of tip RSA (marker 131) and hemisphere RSA (marker 121) in a patient.

We have previously validated the use of marker-free RSA for use in a humeral head resurfacing prosthesis (Sköldenberg et al. 2011), and we used the same type of experimental setting for this study. We used an Episealer implant, and the manufacturer modified the tip of the implant into a 3-mm hemisphere. In addition, we then marked the component with 1 tantalum marker at the tip (Figure 2). The prosthesis was implanted in a knee phantom (Sawbones; Sawbones Europe, Malmö, Sweden) and 6 tantalum markers (1.0 mm) were placed in the sawbone to serve as the reference segment for the RSA analysis. The phantom was then placed in a biplanar calibration cage (Cage 10; RSA Biomedical AB, Umeå, Sweden). Digital radiographs (Bucky Diagnostic; Philips, Eindhoven, the Netherlands) were then taken using 1 fixed and 1 mobile X-ray source. The exposure was set to 125 kV and 2.5 mAs. The radiographs were saved in a standard dicom file format (resolution 254 dpi) and uploaded to a workstation. UmRSA 6.0 computer software (RSA Biomedical) was used for all measurements and migration analyses.

We performed the following procedure to measure the migration of the implant in relation to the sawbone. 1) The knee phantom was placed in the calibration cage at the point of intersection of the central radiograms. 2) 1 set of radiographs was taken (position 1, series 1). 3) The calibration cage, the X-ray tubes, and the phantom were repositioned. 4) 1 set of radiographs was taken (position 1, series 2). 5) The prosthesis was moved 0.5–1.0 mm in relation to the sawbone, to simulate migration of the implant. Steps 1 to 5 were then repeated 20 times, giving us position 2, series 1 and 2, position 3, series 1 and 2 and so on. The markers in the sawbone formed the 3D reference segment and were not altered between exposures.

### Standard RSA

For standard RSA, point motion of the tantalum bead fixed to the tip of the implant was measured using standard automated RSA (Figure 2).

### Tip and hemisphere RSA

We then used 2 different methods of measuring the tip; the most distal part of the hemisphere visible on the radiographs (tip RSA) and the center of the tip hemisphere (hemisphere RSA). These 2 measurements were done manually and fed into the RSA software (Figure 2). 20 repeated stereograms with gradual shifts of position of the specimen between each double exposure were used for the analysis. The tip and hemisphere measurements were then compared to the RSA point motion of the tantalum bead fixed to the tip of the implant to determine the accuracy and precision of the latter.

### Precision

The precision of a measurement, also called the reproducibility or repeatability, is the degree to which repeated measurements under unchanged conditions show the same results, and it refers to random errors only. To calculate the precision of both RSA methods, we analyzed the double measurements (series 1 and 2) taken at each of the 20 positions for migration. The difference between the double measurements was then calculated; for instance, for x-translation (*xt*): *d* *_precxt_* = *xt* *_p1:1_* – *xt* *_p1:2_* where *d* *_precxt _*is the difference between position 1, series 1 (*p* *_1:1_*) and position 1, series 2 (*p* *_1:2_*). Since no migration of the tip of the implant in relation to the sawbone had occurred, this difference represents the precision of the methods. For each of the methods used (standard, tip, and hemisphere), we had 20 double sets of radiographs on which to calculate precision.

### Accuracy

Accuracy is defined as the degree of closeness between a measured value, which has been derived from a series of measurements (in this study, tip and hemisphere RSA), and the true value (the gold standard; in this study, from standard point-motion RSA). Accuracy includes both random and systematic errors. In this laboratory setting, the accuracy of standard point-motion RSA was assumed to be perfect; i.e. standard RSA measures the true migration of the implant (Valstar et al. 2000).

In order to calculate the accuracy of the tip and hemisphere RSA, we measured the migration between 2 phantom positions with both standard RSA and tip and hemisphere RSA. For example, for y-translation (*yt*): *RSA* *_yt1–2_* = *yt* *_p1_* – *yt* *_p2 _*where *RSA* *_yt1–2_* is the migration in y-translation of the prosthesis between position 1 (*p1*) and position 2 (*p2*) measured with standard RSA. *Tip* *_yt1–2 _*and *Hemi* *_yt1–2_* is the same migration measured with tip and hemisphere RSA, respectively. The difference between these measurements was then calculated for y-translation (*yt*): *d* *_accurTipyt _*= *RSA* *_yt1–2 _*– *Tip* *_yt1–2 _*and *d* *_accurHemiyt _*= *RSA* *_yt1–2 _*– *Hemi* *_yt1–2_*. Ideally, these would be zero since all methods measured the same migration. To generate independent measurements, this was calculated pairwise in positions 1–2, 3–4, and 5–6. As 20 different positions were measured, 10 different pairs of migration analyses were performed each to determine the accuracy of tip and hemisphere RSA.

### Statistics

We calculated precision and accuracy for point-motion translations in the x-, y-, and z-translations and also the maximum total point motion (MTPM), which is the length of the 3D vector of the implant marker that moved the most: in this study, the tip of the implant (Ryd et al. 1995). We defined the precision for standard, tip, and hemisphere RSA as 2.09 SD (20 degrees of freedom (d.o.f.)) of the difference between the double examinations (*d* *_prec_*). 2.09 is the 95% quantile for the t-distribution with 20 d.o.f., and this was chosen for precision since only random errors are included in precision measurements. We defined the accuracy for tip and hemisphere RSA as 2.26 RMS (9 d.o.f.) of *d* *_accur_* (“root mean square”, a measure of the magnitude of varying quantity, since the difference between the standard RSA and the 2 manual methods could be both positive and negative). 2.26 is the 95% quantile for the t-distribution with 9 d.o.f., since accuracy involves both systemic and random errors. For all precision and accuracy calculations, we also present the mean and range. We used SPSS software version 20.0 for Macintosh.

## Results

The precision for point motion was similar for tip and hemisphere RSA for translations, ranging between 0.12 mm and 0.33 mm. Precision for total migration (MTPM) was also similar for the 2 manual methods: 0.20 and 0.18 mm (Table 1). The accuracy for tip RSA ranged from 0.08 mm to 0.17 mm and for hemisphere RSA it ranged between 0.09 mm and 0.19 mm (Table 2).

**Table 1.** Precision of standard, tip, and hemisphere RSA

	Standard RSA				Tip RSA				Hemisphere RSA			
	2.09 × SD	mean	min	max	2.09 × SD	mean	min	max	2.09 × SD	mean	min	max
Translation (mm)												
x	0.02	–0.01	–0.05	0.09	0.14	0.07	–0.07	0.19	0.18	0.03	–0.08	0.19
y	0.04	0.02	–0.01	0.07	0.19	0.02	–0.13	0.21	0.12	–0.04	–0.14	0.09
z	0.09	0.01	–0.09	0.12	0.20	0.08	–0.15	0.23	0.33	0.05	–0.25	0.36
Total migration (mm)												
MTPM	0.11	0.14	0.0	0.19	0.20	0.23	0.09	0.46	0.18	0.26	0.08	0.37

**Table 2.** Accuracy of tip and hemisphere RSA

	Tip RSA				Hemisphere RSA			
	2.26 × RMS	mean	min	max	2.26 × RMS	mean	min	max
Translation (mm)								
x	0.10	0.00	–0.11	0.05	0.09	0.02	–0.04	0.09
y	0.08	–0.02	–0.06	0.03	0.17	–0.03	–0.14	0.11
z	0.17	0.03	–0.06	0.15	0.19	0.03	–0.11	0.14
Total migration (mm)								
MTPM	0.20	0.03	–0.06	0.15	0.23	0.09	0.05	0.17

## Discussion

Our aim was to validate manually measured point-motion RSA when used with a modified hemispherical tip instead of a tantalum marker bead. We found that both accuracy and precision were adequate for measurement of translation and total migration (MTPM). Either the tip of the implant or the estimated center of a small hemisphere constructed by machining the tip, can be used to detect small migration.

To our knowledge, there have not been any previous publications on RSA and focal knee resurfacing implants. There have, however, been numerous publications on total knee arthroplasty and RSA. Our precision and accuracy was well within the limit of detecting both large initial migration and continuous migration of more than 0.5 mm, which is predictive of late failure of a knee prosthesis (Ryd et al. 1995).

An alternative RSA method for the current study could have been model-based RSA (Kaptein et al. 2003), where no implant marking is necessary. However, this method requires either computer-aided design (CAD) models or models obtained from reversed engineering (RE) of the actual implant. The method assumes a perfect manufacturing process with identical implants implanted for each component size, and any inaccuracies in the size and/or the surface of the prosthesis will reduce the precision. All patients operated with the new focal knee implant receive custom-made implants, making it time- and resource-consuming to manufacture individual CAD models to be used for each RSA analysis. From the results of the current study, our conclusion is that machining just the tip of the implant is the best way of assuring correct measurement of its migration and translation.

1 previous RSA validation study regarding tip motion of a knee implant has been published. Laende et al. (2009) used the circle-finding feature of the RSA software to mark the tip of a tibial component in total knee arthroplasty. They used the center of the stem tip as an additional implant marker, which improved their marker distribution for better rigid body estimation. The use of this feature, originally intended for identification of the spherical femoral head in total hip arthroplasty (Kärrholm et al. 1997), was not feasible in our study because of the small diameter of the distal tip (3 mm). In another type of RSA study in which non-tantalum markers were used, Madanat et al. (2009) validated radio-opaque bioactive glass markers for radiostereometric analysis in a phantom study on fracture micromotion. They analyzed segment motion and used 1.5-mm spherical markers and the same software as in our study, and found accuracy and precision values ranging between 0.009 mm and 0.05 mm and 0.18–0.87 degrees. They did not specify whether they used the manual or automatic method for marking of the individual markers. Based on their excellent precision, it is likely that they managed to use the automated measuring method and thus reduced the inherent “noise” in doing manual measurements.

When fixating small unipolar focal knee resurfacing (FKR) implants (articulating against opposing cartilage), new challenges are encountered. Large titanium (Ti) anchoring screws used in similar implants (Hemicap) could potentially create large defects upon removal, thus augmenting the complexity of revision surgery. The use of Ti implants has shown satisfactory osseointegration in dental implants where unloading is possible (Adell et al. 1981). Our implant uses a thin double-coated press-fit peg for fixation, which has shown good results in animals (Martinez-Carranza et al 2013) but has so far not been proven in the clinical setting. The current study is thus important for validation of the method to be used in clinical trials in humans.

This study had some limitations. The greatest disadvantages of using point motion is, of course, that segment motion with rotations cannot be evaluated and that we are using the motion of the tip of the implant as a proxy for the overall migration of the implant. However, because of the small size of the implant, marking with tantalum beads would be hazardous for the integrity of the component-making standard. Thus, segment-motion RSA with full evaluation of rotation of the implant is not possible.

In addition, no method with better accuracy than standard RSA was available with which to verify the difference between standard RSA and the manual methods. In phantom studies like this, however, the accuracy of standard RSA is very close to perfect (Valstar et al. 2000), and as we only intended to describe the migration in a phantom model, we consider that for all practical purposes this assumption is correct. The good precision of standard RSA for translations and MTPM in the present study (Table 1) strengthens this hypothesis. Also, we had no access to cadaver knees in which to implant the prosthesis. Thus, the effect of bone and soft tissue in reducing the precision could not be accounted for. As in all RSA studies, it is therefore important to perform double measurements when using this type of manual RSA in clinical trials (Valstar et al. 2005).

In conclusion, the accuracy and precision for point motion is acceptable when using a small hemisphere at the tip of a focal articular knee resurfacing implant instead of tantalum marker beads. The method is accurate and precise enough to detect relevant migration, and it will be used for future clinical trials with the new implant.
